# Prevalence of *DICER1* variants in large multinodular goiter: thyroid function, clinical and imaging characteristics

**DOI:** 10.20945/2359-4292-2023-0030

**Published:** 2024-02-08

**Authors:** Lara Judith Cabral Miranda, Débora L. S. Danilovic, Felipe Augusto Brasileiro Vanderlei, Marcos Roberto Tavares, Nicolau Lima, Rosalinda Yossie Asato de Camargo, Suemi Marui

**Affiliations:** 1 Universidade de São Paulo Faculdade de Medicina Hospital das Clínicas São Paulo SP Brasil Laboratório de Endocrinologia Celular e Molecular (LIM25), Hospital das Clínicas, Faculdade de Medicina, Universidade de São Paulo, São Paulo, SP, Brasil; 2 Universidade de São Paulo Faculdade de Medicina Hospital das Clínicas São Paulo SP Brasil Unidade de Tireoide, Disciplina de Endocrinologia e Metabologia, Hospital das Clínicas, Faculdade de Medicina, Universidade de São Paulo, São Paulo, SP, Brasil; 3 Universidade de São Paulo Faculdade de Medicina Hospital das Clínicas São Paulo SP Brasil Departamento de Cirurgia, Disciplina de Cirurgia de Cabeça e Pescoço, Hospital das Clínicas, Faculdade de Medicina, Universidade de São Paulo, São Paulo, SP, Brasil

**Keywords:** Goiter, genetics, DICER1, thyroid nodules

## Abstract

**Objective:**

Mutations in DICER1 are found in differentiated thyroid carcinoma (DTC) and in multinodular goiter (MNG) at a younger age with other tumors, which characterizes DICER1 syndrome. DICER1 is one driver to DTC; however, it is also found in benign nodules. We speculated that patients with mutations in DICER1 may present long-lasting MNG. Our aim was to investigate the frequency of DICER1 variants in patients with MNG.

**Subjects and methods:**

Patients who submitted to total thyroidectomy due to large MNG with symptoms were evaluated. DICER1 hotspots were sequenced from thyroid nodule samples. To confirm somatic mutation, DNA from peripheral blood was also analyzed.

**Results:**

Among 715 patients, 154 were evaluated with 56.2 ± 12.3 years old (28-79) and the thyroid volume was 115.7 ± 108 mL (16.2-730). We found 11% with six DICER1 variations in a homo or heterozygous state. Only rs12018992 was a somatic DICER1 variant. All remaining variants were synonymous and likely benign, according to the ClinVar database. The rs12018992 was previously described in an adolescent with DTC, measuring 13 mm. There were no significant differences according to gender, familial history of goiter, age, thyroid volume, TSH and TI-RADS classification between DICER1 carriers. Free T4 were lower in patients with DICER1 polymorphisms (13.77 ± 1.8 vs. 15.44 ± 2.4 pmol/L, p = 0.008), regardless of TSH levels.

**Conclusions:**

We conclude that germline *DICER1* variants can be found in 11% of large goiters but no second-hit somatic mutation was found. DICER1 is one driver to thyroid lesion and a second-hit event seems unnecessary in the MNG development.

## INTRODUCTION

Multinodular goiter (MNG) is a common disease, found in up to 20% of the population, being more frequent in women and older patients and in iodine insufficient regions (1). Chronic iodine deficiency is a well-known etiology of MNG that leads to a compensatory increase in thyroid follicular cells, nodules development and it increases the total volume of the gland (1).

However, in the presence of iodine sufficiency, what causes MNG is still unknown. Somatic mutations in the TSH receptor (*TSHR*) have been identified as causing clonal or polyclonal cell proliferation, and consequently autonomous nodules as they are not responsive to endogenous TSH (2).

Through linkage studies, the first associated *locus* was *MNG-1*, located on chromosome *14q31*, identified in a Canadian family with 18 affected members with nontoxic MNG, in which 2 individuals also had papillary thyroid carcinoma (3). Another *locus*, called *MNG-2* on chromosome *Xp22* was also associated with MNG (3).

More recently, MNG was found in patients with DICER1 syndrome, an autosomal dominant disease, with variable penetrance, which increases the predisposition to the development of malignant and benign tumors, whether in childhood or adulthood (4). DICER1 syndrome is characterized by the presence of pleuropulmonary blastoma and embryonic cervical rhabdomyosarcoma (OMIM 601200). Furthermore, pituitary blastoma in children, cystic nephroma, ovarian sexual cord stromal tumors and ciliary body medulloepithelioma have also been found (5). Interestingly, MNG was present, associated with papillary thyroid carcinoma, with a high penetrance especially in women (OMIM 606241) (5).

DICER1 is an essential RNase-III endonuclease for processing pre-miRNA into mature, functional miRNA (4), and the gene is located on chromosome *14q32.13,* the locus is the so-called *MNG-1*. The DICER1 protein has two catalytic RNase-III domains, IIIa and IIIb. In more than 50% of cases, germline *DICER1* mutations result in truncated proteins, while second-hit somatic mutations are typically missense mutations at the IIIa/IIIb metal binding sites, affecting four amino acids of the RNase-IIIB domain (6,7).

Wasserman and cols. described somatic and germline *DICER1* mutations in young patients (<18 years old) submitted to thyroidectomy, without common tumoral development of the DICER syndrome, characterizing one more candidate gene involved in the pathophysiology of thyroid cancer, specifically papillary carcinoma (8,9). In contrast, somatic and germline *DICER1* mutations have also been identified in 20% of patients with benign thyroid nodules, with a histology of hyperplastic follicular nodules with papillary growth, measuring 20 mm to 28 mm (8). Additionally, in most *DICER1*-associated tumors, there are two hits in *DICER1* somatically acquired, whereas *DICER1*-associated benign nodules carry a germline truncating mutation and an additional somatic mutation by a second hit, which may suggest an occult DICER1 syndrome in adults with thyroid nodules (10,11).

We speculated that patients with somatic mutations in *DICER1* may present long-lasting MNG that is not associated with thyroid cancer or other neoplasms. Thus, this study aimed to investigate the frequency of *DICER1* gene variants in patients with MNG and to correlate with clinical findings.

## SUBJECTS AND METHODS

A retrospective study was conducted to include patients who were submitted to total thyroidectomy from 2011 to 2019 due to a multinodular goiter at a tertiary care hospital (*Hospital das Clínicas* – HCFMUSP). Patient data and outcomes were collected through a retrospective chart review. The Research Ethics Committee of the University of Sao Paulo (46329221.6.0000.0068) approved the study.

### Inclusion criteria

All patients with a multinodular goiter with surgical indication of total thyroidectomy because of tracheal deviation, substernal goiter or thyroid nodule > 4 cm were included.

### Exclusion criteria

Patients under 18 years old or on treatment with drugs that induce thyroid diseases (i.e., amiodarone, lithium, interferon, iodine) were excluded. Patients with previous thyroidectomy and radioactive iodine treatment were also excluded. Patients with autoimmune thyroid disease determined by the presence of anti-thyroperoxidase antibodies (TPOAb, Hashimoto's thyroiditis) or antibody against the TSH receptor (TRAb, Graves' disease), and patients with histology records of thyroid adenoma or carcinoma (papillary and follicular) were all excluded. Additionally, patients with a familial history of goiter and clinical features of DICER1 syndrome (such as a past diagnosis of lung tumor, rhabdomyosarcoma, pituitary lesions, kidney or ovary tumors), or a first-degree relative with DICER1 syndrome were excluded to avoid finding well-known *DICER*1 variations.

### Subjects

Clinical characteristics, demographic data, laboratory results and histological features were retrospectively collected.

The presence of a substernal goiter and tracheal deviation were diagnosed by computed tomography and/or chest X-ray. The following thyroid nodule characteristics were determined by ultrasound as defined by the Thyroid Imaging Reporting and Data System (TI-RADS) (12): echogenicity, composition, margins, shape and presence of echogenic foci. A fine needle aspiration biopsy (FNA) was performed as the ACR-TIRADS and ATA guideline suggested (13). Bethesda system was reported as previously described (14).

Serum TSH (normal range NR 0.27-4.20 mU/L), free T4 (FT4) (NR 9.0-21.8 pmol/L) and/or total T3 (1.22-2.76 nmol/L) were measured by commercial kits (Roche Diagnostics, Germany) within 6 months prior to surgery. Thyroid status was designed as euthyroidism when TSH and FT4 were within normal ranges, hypothyroidism with TSH > 4.20 mU/L with low or normal FT4 (subclinical hypothyroidism), hyperthyroidism with TSH < 0.27 mU/L with high or normal FT4 and/or total T3 (subclinical hyperthyroidism).

### DNA extraction and sequencing

Fresh tissue thyroid samples (~0.5 cm) were collected in the operating room from the largest nodule and immediately cryopreserved until DNA extraction. The remaining thyroid gland was formalin-fixed and paraffin-embedded and submitted to a histological evaluation by HCFMUSP Pathology Service. DNA was extracted from fresh specimens using QIAGEN AllPrep DNA/RNA Mini Kit (QIAGEN, Hilden, Germany) followed by purification according to the manufacturer's instructions.

*DICER1* hotspot regions corresponding to RNAse IIIa and IIIb domains (including exons 19 to 26) were amplified by PCR and directly sequenced in an ABI Prism Genetic Analyzer 3130xl automatic DNA sequencer (Applied Biosystems, Foster City, USA) as previously described (7) and compared to the genomic sequence provided by Ensembl Genome Browser (ENSG00000100697). *DICER*1 variants were searched in ClinVar (https://www.ncbi.nlm.nih.gov/clinvar/) to evaluate their significance to disease.

DNA was extracted from peripheral blood using QIAGEN Gentra Puregene Blood Kit (Qiagen, Hilden, Germany) according to the manufacturer's instructions and *DICER1* hotspot regions were amplified as described above to confirm germline mutation.

### Statistical analysis

Data were processed using IBM SPSS Statistics for Windows version 26 (IBM, Armonk, NY). Categorical variables are presented as absolute and relative frequencies. Differences were evaluated by Pearson's chi-square test and Fisher's exact test when appropriate. Continuous variables are presented as mean ± SD or median (range). Differences among studied subgroups were determined using the Student's t-test if presenting normal distribution, and the Mann-Whitney U test for non-normal distributions. Two-tailed p values were used and p values < 0.05 were considered statistically significant.

## RESULTS

**Figure 1:**
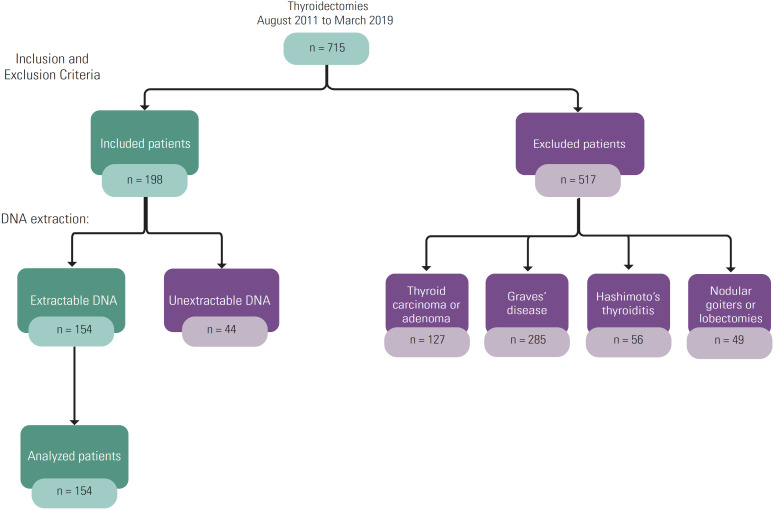
Summary of participants. The flowchart shows the distribution of research participants.

From a total of 715 patients submitted to total thyroidectomy between 2011 and 2019 (Figure 1), we evaluated 154 patients (92% female) with a mean age of 56.2 ± 12.3 years (28–79 years). Two years was the mean time between onset of symptoms (dyspnea and/or difficult to swallow) and thyroidectomy.

Mean thyroid volume was 115.7 ± 108.0 mL (16.2-730 mL), being 84% in euthyroidism status (Table 1). Tracheal deviation was present in 105 patients (68%) and a substernal goiter was diagnosed by computed tomography in 72/101 patients. Positive correlation between thyroid volume and tracheal deviation as well as the presence of a substernal component were observed (p < 0.001). Thyroid volume was also associated with symptoms, such as dyspnea and/or difficulty to swallow and older age at thyroidectomy (p < 0.001).

**Table 1:** Thyroid status of all patients submitted to total thyroidectomy

	Presence of *DICER1* variants	Absence of *DICER1* variants	Total
Hyperthyroidism (TSH < 0.27 mU/L and FT4 > 21.9 pmol/L or T3 > 2.76 nmol/L)	0	3	3 (1.9%)
Subclinical hyperthyroidism (TSH < 0.27 mU/L and FT4 9.0-21.8 pmol/L)	0	20	20 (12.9%)
Euthyroidism (TSH 0.27-4.20 mU/L)	17	113	130 (83.9%)
Subclinical hypothyroidism (TSH > 4.20 mU/L and FT4 9.0-21.8 pmol/L)	0	1	1 (0.7%)
Total	17 (11%)	137 (89%)	154 (100%)

FT4: free T4.

TI-RADS classifications of thyroid nodules were TR2 to TR4 in 129 nodules and FNA was performed in 109 nodules (71%) with Bethesda cytology II to V in 88%, 10%, 1% and 1%, respectively (Table 2). Histopathological analyses report a multinodular goiter or an adenomatous goiter with adenomatoid nodular hyperplasia in all, such as thyroid adenoma or carcinoma as well as autoimmune thyroid disease were excluded from molecular analysis.

**Table 2:** ACR TI-RADS classification according to the presence of DICER1 variants

	ACR TI-RADS classification		
	2	3	4
Presence of *DICER1* variants	0	4	2
Absence of *DICER1* variants	75	39	9

ACR TI-RADS classification was determined according to ultrasound characteristics (13).

We identified 6 polymorphisms in the *DICER1* gene previously described in exons 19, 20, 23 and 24, affecting 17 patients with thyroid nodules (11% of total patients), in a homo or heterozygous state (Table 3). All were synonymous variants. All but one patient harbored germline variants, which the presence of the same variant in the peripheral blood DNA analysis confirmed. From the 17 patients with variants in the *DICER1* gene, nine thyroid nodules had only one variant, while 8 showed 2 variants. The most found variant was c.5241G>A, p.Ser1747= (rs114861074), detected in 9 thyroid nodules, commonly found associated with c.3033G>A, p.Ala1011= (rs8019857). Other variants found were c.2997T>G, p.Leu999= (rs12018992), c.4680G>A, p.Ala1560= (rs61729797), c.4515T>C, p.Ser1505= (rs141308332) and c.3198T>C, p. Thr1066= (rs114964211) (Table 3).

**Table 3:** *DICER1* variants found in patients submitted to total thyroidectomy

Case	Variant	Ensembl code	State
1	c.5241G>A, p.Ser1747=	rs114861074	Heterozygous/Germline
2	c.2997T>G, p.Leu999=	rs12018992	Heterozygous/Germline
3	c.3033G>A, p.Ala1011= and c.4680G>A, p.Ala1560=	rs8019857 and rs61729797	Both heterozygous/Both germline
4	c.3198T>C, p.Thr1066= and c.5241G>A, p.Ser1747=	rs114964211 and rs114861074	Both heterozygous/Both germline
5	c.4515T>C, p.Ser1505=	rs141308332	Heterozygous/Germline
6	c.2997T>G, p.Leu999=	rs12018992	Heterozygous/Germline
7	c.3033G>A, p.Ala1011= and c.5241G>A, p.Ser1747=	rs8019857 and rs114861074	Both heterozygous/Both germline
8	c.3033G>A, p.Ala1011= and c.5241G>A, p.Ser1747=	rs8019857 and rs114861074	Both heterozygous/Both germline
9	c.3033G>A, p.Ala1011= and c.5241G>A, p.Ser1747=	rs8019857 and rs114861074	Both heterozygous/Both germline
10	c.2997T>G, p.Leu999=	rs12018992	Heterozygous/Germline
11	c.3033G>A, p.Ala1011= and c.5241G>A, p.Ser1747=	rs8019857 and rs114861074	Both heterozygous/Both germline
12	c.3033G>A, p.Ala1011= and c.5241G>A, p.Ser1747=	rs8019857 and rs114861074	Both heterozygous/Both germline
13	c.5241G>A, p.Ser1747=	rs114861074	Heterozygous/Germline
14	c.3033G>A, p.Ala1011=	rs8019857	Heterozygous/Somatic
15	c.3033G>A, p.Ala1011=	rs8019857	Homozygous/Germline
16	c.3033G>A, p.Ala1011= and c.5241G>A, p.Ser1747=	rs8019857 and rs114861074	Both heterozygous/Both germline
17	c.2997T>G, p.Leu999=	rs12018992	Heterozygous/Germline

We searched all variations found in our samples in ClinVar, a freely accessible, public archive of reports of human genetic variants and interpretations of their significance to disease, maintained by the National Institutes of Health (https://www.ncbi.nlm.nih.gov/clinvar/). ClinVar aggregates data by variant-disease pairs and by variant (or set of variants). There are no citations in ClinVar for all variations except for rs12018992 (15–20). Patients 10 and 17 presented the rs12018992 in a heterozygous state with benign thyroid nodules measuring 3.4 and 7.0 cm with a thyroid total volume of 55.5 and 42.7 mL.

## DISCUSSION AND CONCLUSION

In this study, we report the prevalence of the *DICER1* gene variants in adult patients with multinodular goiter submitted to a total thyroidectomy due to a large goiter. No *DICER1* mutation was found in the thyroid nodules. We found synonymous polymorphisms in the *DICER1* gene in 11% of patients, all considered benign. There are no citations in ClinVar for any variations except for rs12018992 (15–20). Although there is no functional evidence, all variants can be considered likely benign or benign based on the following criteria: it is a conservative change, it occurs at a poorly conserved position in the protein and it is predicted to be benign by multiple *in silico* algorithms (15–20). Only the rs12018992 was previously found in one patient diagnosed at 15 years old with a minimally invasive solid variant of papillary thyroid carcinoma, measuring 13 mm (8). The patient had normal thyroid function and a familial history of goiter. The variant was found as a germline variant in a heterozygous state with no somatic finding and showed no evidence of a second hit within the *DICER1* locus (8). Patients 10 and 17 presented the rs12018992 in a heterozygous state. They were both female, 52 and 55 years old, with thyroid nodules measuring 3.4 and 7.0 cm and thyroid total volume of 55.5 and 42.7 mL and with normal thyroid function.

Patients with germline *DICER1* pathogenic variants are at risk of developing thyroid nodules and a multinodular goiter, particularly in young age (21), whereas in adult-onset familial multinodular goiter is considered rare (5). In a previous study, 1.4% of adult onset of thyroid nodules harbor a hotspot *DICER1* mutation (10). Samples were obtained from FNA with Bethesda II to V results, with *DICER1* mutations first identified by ThyroSeq v3 (214 from 14,993 samples). The thyroid nodule size was not referred to. Then, they searched for alterations in the full coding region of *DICER1* in only 60 samples and three-quarters of these mutations were accompanied by a second, likely loss of a function "hit" in *DICER1* (10). Hyperplastic thyroid nodules were diagnosed in only 3 patients because follow-up was obtained in very few cases (8 out of 60 patients). We speculated that *DICER1* mutations were likely to drive these thyroid nodules in adult patients, which they considered mostly benign. Some of these loss-of-function variants are probably germline in origin, thus representing previously undiagnosed DICER1 syndrome patients or atypical presentation of DICER1 syndrome. They concluded that identification of germline pathogenic variants in adults would have a direct effect on the clinical management of the patients and they would serve as the gateway to identify relatives who are at risk of pediatric malignancies (10).

In our study, we selected only patients with no clinical characteristics of DICER1 syndrome with large benign thyroid nodules. We expected that somatic mutations would be found in sporadic benign thyroid tumors causing large goiters without clinical characteristics of DICER1 syndrome. The Cancer Genome Atlas project identified pathogenic somatic *DICER1* variants in 0.5% of papillary thyroid cancer and almost 50% in pediatric-onset thyroid neoplasia (22). We only found highly benign *DICER1* germline variants in large thyroid nodules, not cancer, and the variants' role in thyroid hyperplasia and extensive follicular growth is unknown. Whereas we did not find the second-hit mutation or even a somatic pathogenic mutation in *DICER1*; germline *DICER1* variants possibly only drive hyperplasia but not thyroid cancer. The frequency of *DICER1* variants in sporadic multinodular goiter with adult onset is still unknown, but we found a much higher (11%) than previously reported in adult-onset thyroid cancer (10,11). Because we only found nonpathogenic *DICER1* variants, other causes of a multinodular goiter must be involved in adult sporadic long-lasting presentation, such as iodine and selenium deficiencies or variants in other genes involved in thyroid development but not causing hypothyroidism. We also only searched the hotspots region (exon 19 to 26), located within the RNase IIIB domain of *DICER1*, that are likely to affect the function of DICER1. In all cases where the entire *DICER1* gene could be examined, the *DICER1* mutation appeared to be present in tandem with hotspot mutations (23). Therefore, finding mutations in other regions of the gene is unlikely.

Iodine status plays a major role in the development of thyroid nodularity and goiter, and iodine deficiency is the most important environmental factor that increases the risk for development of endemic and sporadic goiter (24). Our study did not evaluate iodine intake status, which should be considered an extrinsic factor that contributes to the development of goiter. Since 1953, salt iodization has been mandatory in Brazil, which was previously categorized as a "more than adequate" category (until 2015). It is now reported as an iodine-intake-adequate area, according to Iodine Global Network (http://www.ign.org) (25).

Selenium deficiency may be involved in the pathogenesis of multinodular goiter because selenium also plays an important role for normal thyroid function and development (26). Selenium deficiency is commonly seen in an iodine deficiency area, and an accurate diagnosis of deficiency and consequently its treatment are still not widely recommended.

Besides micronutrients deficiencies, TSH is the most important growth factor involved in the pathogenesis of a goiter (26). However, we selected patients with normal TSH levels, and suppression of TSH may not stop thyroid growth. Therefore, other growth factors are also implicated in the goiter's development, such as an insulin-like growth factor and a transforming growth factor (TGF) (26).

Genes involved in thyroid hormone synthesis, such as thyroglobulin, TPO, TSH receptor and solute carrier family and dual oxidase 2, are also candidate genes for goiters with hypothyroidism. Few cases were described with euthyroidism and goiter due to a combined defective step in thyroid hormone synthesis (27). Differential gene expression studies in a non-functioning thyroid nodule in an endemic goiter contributes to the understanding of the genesis of multinodular goiter and a possible molecular marker, such as the HOTS gene (H19 opposite tumor suppressor) (28) and cellular fibronectin (29).

Unfortunately, we did not classify functioning and non-functioning thyroid nodules in our cohort, which might have differential molecular pathogenesis. However, at clinical practice, determination of thyroid function using radioiodine scan, is only performed with suppressed TSH. Most goiters present normal radioiodine uptake with functioning and non-functioning nodules in the same gland.

In our cohort, patients with *DICER1* variants showed, before surgery, a lower concentration of FT4 within the normal range (13.77 ± 1.8 pmol/L *vs.* 15.44 ± 2.4 pmol/L, p = 0.008) with TSH levels showing no difference. Dicer knockout mice develop severe hypothyroidism due to failure of thyroid gland follicular architecture in later life (30). FT4 and TSH concentrations were previously reported and were also within normal range in younger patients with *DICER1* mutations, but they were not compared to the control group (8). Until now, DICER1 has not been related to deiodinase activity or other thyroid function genes. It is premature to speculate our findings that *DICER1* variants could be involved in FT4 set point because low FT4 concentrations stimulate TSH secretion, which is a well-known thyroid follicular stimulatory growth factor, promoting hyperplasia, thyroid nodules and finally, huge goiter. Nevertheless, patients harboring the *DICER1* variants showed normal TSH levels in our cohort (mean 1.18 ± 0.72 mU/L).

We conclude that germline *DICER1* variants can be found in 11% of large goiters but no second-hit somatic mutation was found. DICER1 is one driver to thyroid lesion and a second-hit event seems unnecessary in the MNG development.
